# Elevating the Role of School Nurses in School-Based Mental and Behavioral Health: A Consensus Document

**DOI:** 10.1177/1942602X241295803

**Published:** 2024-11-13

**Authors:** National Association of School Nurses

**Keywords:** Collaboration, Multidisciplinary Teams, Mental Health, Behavioral Health, School-based Mental Health

## Abstract

School nurses are members of the school-based mental and behavioral health support team. This consensus document was developed by school nurses and school nurse leaders in collaboration with leaders from national associations and organizations with a vested interest in K-12 mental and behavioral health. The document is also publicly available on the National Association of School Nurses website.

## Introduction

According to a recent report from the Centers for Disease Control and Prevention (CDC), the number of adolescents experiencing poor mental health is growing, especially among LGBTQ+, female, and Black students (CDC, 2023). In 2021 alone, 70% of LGBTQ+ students reported persistent feelings of hopelessness, and 25% of female students had made a suicide plan. Youth who have mental health problems are more likely to experience violence, engage in risky behavior, and struggle with school. However, CDC notes that when youth have strong feelings of connectedness with school and family, those bonds can protect their mental health. Given the rise in poor mental health among students—and the role school connectedness plays in protecting mental health—it is critical that schools be equipped to provide a safe environment with mental health supports.

To identify key priorities and opportunities in this area, we gathered evidence from the literature and interviewed school health care providers and experts in school-based health services. Ultimately, our goal was to help schools build strong connections with students and deliver school-based mental and behavioral health (SBMH) supports to meet the growing need for them. SBMH care teams manage mental and behavioral health supports in their schools, addressing student needs ranging from stress management, safety planning, and substance use to suicide prevention and coping skills.

## Supporting Youth Mental and Behavioral Health in Schools

Schools play a vital role in preventing mental health problems among youth, as well as in identifying and supporting youth who do have mental health problems. SBMH providers—including social workers, psychologists, counselors, and school nurses as specialized instructional support personnel—lead the way in helping schools fulfill this role. Multi-tiered systems of support (MTSS) is a common framework used by SBMH providers to assist students and match them to appropriate supports. Another framework, positive behavioral interventions and supports, provides a similar tiered approach to organizing and delivering services. Regardless of the framework, many schools have launched a student mental health program, and research shows that these programs help students achieve academically and build social and leadership skills, self-awareness, and positive connections to adults in their school (Youth.gov, n.d.).

Adolescents say they are more comfortable accessing health services through school-based clinics

The rising number of students who have mental health concerns means that schools must prioritize time and resources when considering a student mental health program. Whether schools are just beginning to develop such a program or are seeking to expand an existing one, the SBMH providers we interviewed identified several key areas that schools can focus on to better support students (Box 1).

**Box 1.**
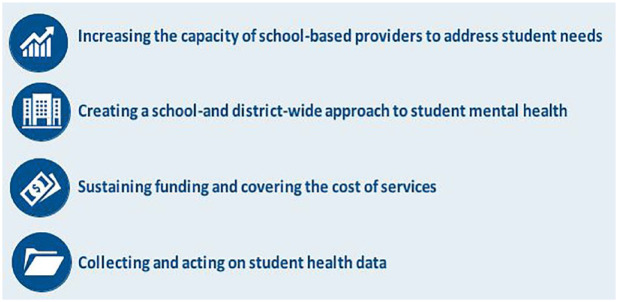
Priority Areas for Advancing SBMH Programs for Students

## How School Nurses can Support SBMH Programs and Teams

School nurses have tremendous potential to work with other SBMH providers to implement school mental health programs, yet they often do not get the opportunity. Although individual school nurses and education systems have different levels of readiness or capacity to support SBMH programs, school nurses are most successful when the SBMH care team, principals, and superintendents engage with them and understand their role.

School nurses can build their SBMH team’s capacity to deploy a multitiered system of supports that is responsive to student needs. As members of interdisciplinary teams, school nurses can collaborate with school personnel—including teachers, psychologists, social workers, counselors, and principals—to assess, identify, conduct interventions for, refer, and follow-up with children who need behavioral health services (Reaves et al., 2022; Substance Abuse and Mental Health Services Administration & Centers for Medicare & Medicaid Services, 2019).

**Image 1.**
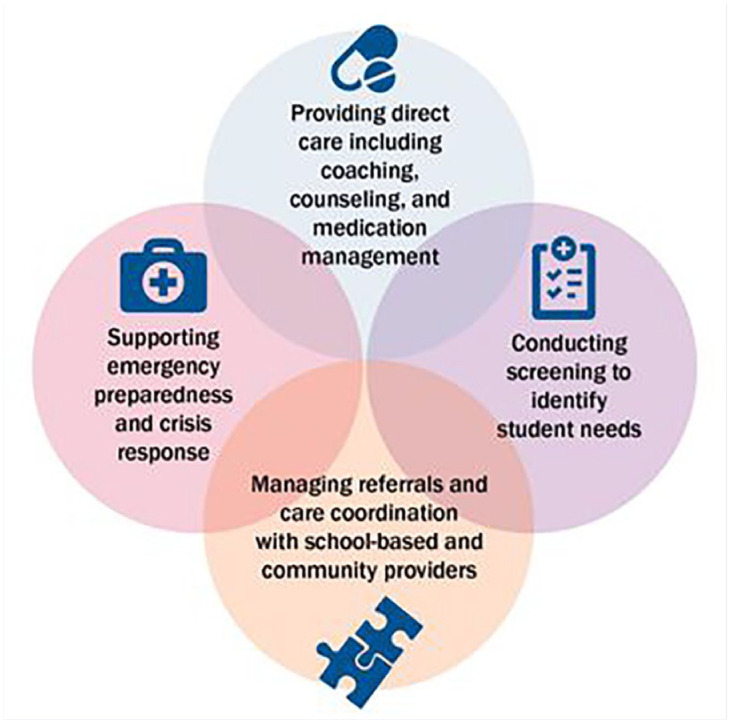
Ways School Nurses Serve as Members of the SBMH Care Team

School nurses are key members of SBMH care teams and support mental and behavioral health (see Image 1). They [school nurses] also have regular access to students in school clinics and are well-positioned to identify students with potential mental health concerns, especially for “frequent visitors”—children who often present with a physical complaint, such as an upset stomach, but could have an underlying mental health issue. This touch point can increase the speed at which problems are identified and treatment can begin.

School nurses promote health by helping students access physical, mental, and behavioral health care in school and in the community (Hoskote et al., 2023). These emerging practices can help policymakers, school administrators and teachers, and SBMH providers think about what school nurses can contribute to their own schools and how they can advance key SBMH priorities (Box 2).

**Box 2.**
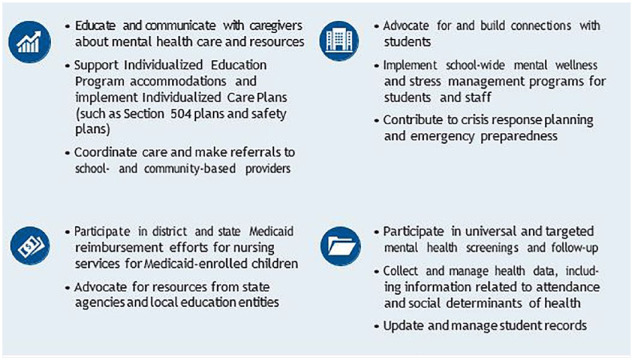
Additional Opportunities for School Nurses to Support SBMH Programs

## Opportunities for Advancing SBMH Programs

The protective effects of school connectedness on youth mental health firmly place SBMH providers, school nurses, and other school personnel on the front lines of responding to the growing crisis (CDC, 2022). However, SBMH providers including school nurses require the broader support of federal and state policymakers, local educational entities, families, and communities to fully realize their opportunity to help students who have mental health problems and those at risk.

The data we gathered from literature reviews and interviews highlight opportunities for state, regional, and community stakeholders to further embed school nurses into existing student support structures, amplify current efforts to address students’ mental and behavioral health needs, and improve systems of care. Box 3 shows opportunities for administrators and policymakers to make substantive changes at the state and regional levels, as well as actions providers and educators at the local and community level can take to support student mental health. We acknowledge that each school nurse and education system might have different level of readiness or capacity to support mental and behavioral health. Nevertheless, adopting some or all the following recommendations can increase system-wide capacity and create a more capable, integrated, holistic, and interconnected system of care.

**Box 3.**
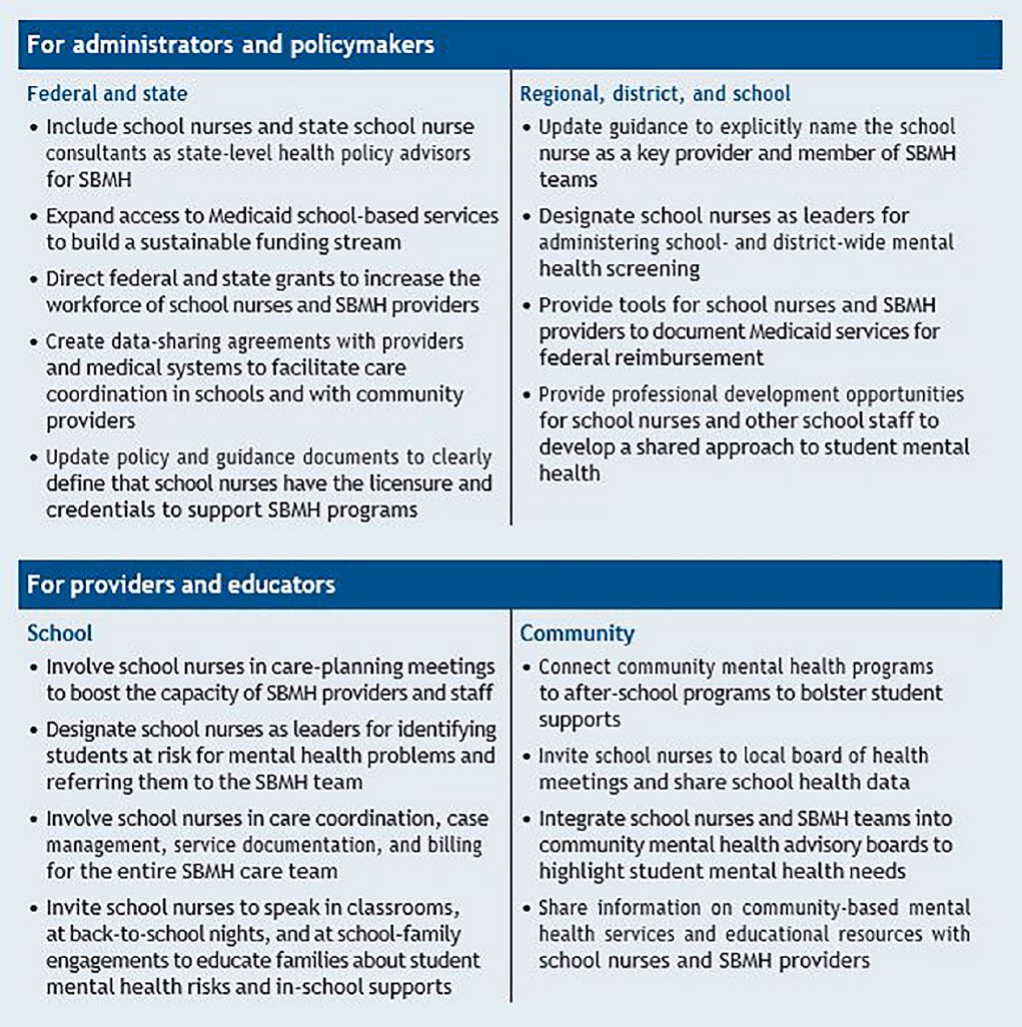
Considerations for Leaders in Health and Education Fields

## Methods and Approach

We completed a literature scan to identify (1) current SBMH models and (2) descriptions of the school nurse’s role in supporting student behavioral and mental health. We analyzed data for common themes, existing programs, opportunities, and challenges to integrating school nurses into SBMH teams.

We held two meetings to discuss the role of school nurses in SBMH care teams and opportunities for further support and integration. The first meeting included school nurses in K–12 schools and school nurse leaders from regional and state-level educational entities. The second meeting involved leaders from national organizations with a vested interest in K–12 mental and behavioral health, including alliances, associations, and councils representing stakeholders from health- and education-based organizations. We analyzed data from these meetings to identify opportunities at the system, community, and school levels to strengthen how school nurses work with other providers to support students’ mental and behavioral health, with an eye toward further integrating school nurses in existing structures.

**Note:** In this document, the terms mental health and mental and behavioral health are used interchangeably to refer to the supports that school nurses provide in mental health and behavioral health, including substance use.

This document is publicly available on the NASN website at: http://higherlogicdownload.s3.amazonaws.com/NASN/8575d1b7-94ad-45ab-808e-d45019cc5c08/UploadedImages/PDFs/Advocacy/CDC-03_SBMH_Consensus_Document_edited_6-14-23-2.pdf
